# Massive Fecal Peritonitis Caused by Stercoral Sigmoid Colonic Perforation in the Elderly

**DOI:** 10.5334/jbr-btr.832

**Published:** 2015-09-15

**Authors:** B. Coulier, O. Tilquin, A. Ramboux

**Affiliations:** 1 Department of Diagnostic Radiology, Clinique St Luc, Bouge (Namur), Belgium; 2 Department of Emergency, Clinique St Luc, Bouge (Namur), Belgium; 3 Department of Visceral Surgery, Clinique St Luc, Bouge (Namur), Belgium

An 83-year-old patient with a recent history of constipation was referred to the emergency room with diagnosis of acute abdomen. The patient had experienced a very acute periombilical pain a few hours before admission. At arrival the pain was continuous and diffuse. Diffuse tenderness with rebound was present especially in the left iliac fossa. The patient was afebrile. Except a mild hyperleucocytosis there was no inflammatory syndrome in blood tests.

Unenhanced Abdominal CT was immediately performed (A = axial view, B = coronal oblique MPR, C = sagital oblique MPR). Diffuse pneumoperitoneum was found with countless small gas bubbles in all peritoneal recesses (black arrow). There was virtually no peritoneal fluid but diffuse infiltration of the peritoneal folds was already present evocating diffuse peritonitis.

Solid feces were still found in the middle portion of the sigmoid colon (black star) and in the rectum but the major part of the entire colon appeared nearly almost flat and empty.

The major CT finding was a very large accumulation of solid feces within the free peritoneal cavity (white stars) extending from the right paraumbilical area to the Douglas’ pouch and that all along the antimesenteric border of the thin and stretched distal sigmoid (white arrows). The level of perforation was not clearly visible. The patient underwent emergency laparotomy. A large ischemic ulcer was found at the level of the rectosigmoid junction and an enormous amount of feces were evacuated. A Hartmann’s procedure was performed and extensive peritoneal lavage was performed with 15 liters of hydrosaline solution.

## Comment

Colon perforation is a rather uncommon event usually caused by malignancy, amoebic colitis, diverticular disease, spontaneous perforation, foreign body perforation, steroid therapy, trauma, and ulcerative colitis. Stercoral ulcer with perforation is a rather very rare event. Fewer than 150 cases have been reported in the literature through 2011 but the incidence is probably underestimated. Severe chronic constipation is considered to be the main causative factor of stercoral colonic perforation because it may induce the formation of stone-hard fecalomas and maintain a persistent pressure over the bowel wall leading to pressure necrosis of the mucosa. Fortunately stercoral ulceration of colonic mucosa does not always occur in the vast majority of constipated patients and not every stercoral ulceration results in colon perforation. A great majority of patients remain at the intermediate state of stercoral colitis (a form of localized ischemia of the colon due to increased intraluminal pressure). The CT findings of stercoral colitis include focal colonic or rectal wall thickening involving the dilated sigmoid colon and rectum, which demonstrate fecalomas. Pericolonic and/or perirectal fat stranding is usually seen due to colonic ischemia or wall edema. The presence of intramural or extraluminal bubble of gas or of an abscess suggests that perforation has already occurred. The three most common locations for stercoral ulceration are the anterior rectum just proximal to the peritoneal reflection, the antimesenteric border of the rectosigmoid junction (the most common site) and the apex of the sigmoid colon. There are several combined reasons why the perforation sites are mainly located in the antimesenteric border of the distal sigmoid colon. First the sigmoid is the narrowest region of the entire colon, and the passage of stools with a more solid consistency at this distal level can be difficult. In such circumstances, fecaloma may exert localized pressure on the walls of the sigmoid colon, particularly in the area with the most precarious vascular supply known as the Sudeck’s critical point (the site of watershed between the supply of the sigmoid arteries and that of the superior rectal artery which is situated at the level of the rectosigmoid junction). Finally prolonged localized pressure and ischemia can give rise to pressure ulceration eventually complicated by stercoral perforation. The mortality related to colon stercoral perforation has been reported to be high. A more favorable outcome in the treatment of stercoral perforation depends upon an immediate treatment of any underlying sepsis, the removal of all stercoral ulcerated diseased colonic tissue, extensive peritoneal lavage, aggressive therapy to counteract colonic perforation peritonitis and appropriate treatment of any co-morbid medical conditions.

**Figures A–C:**
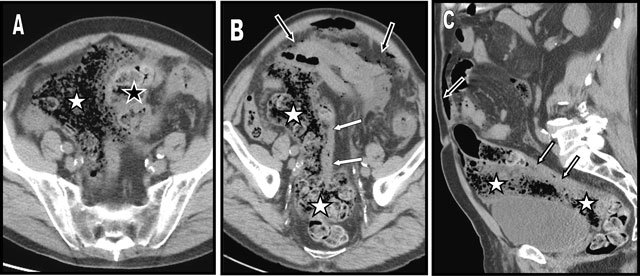


## Competing Interests

The authors declare that they have no competing interests.
